# Diabetes is accompanied by changes in the levels of proteins involved in endosomal GLUT4 trafficking in obese human skeletal muscle

**DOI:** 10.1002/edm2.361

**Published:** 2022-08-14

**Authors:** Rachel Livingstone, Nia J. Bryant, James G. Boyle, John R. Petrie, Gwyn W. Gould

**Affiliations:** ^1^ Institute of Cardiovascular and Medical Sciences University of Glasgow Glasgow UK; ^2^ Institute of Molecular Cell and Systems Biology University of Glasgow Glasgow UK; ^3^ Department of Biology University of York York UK; ^4^ School of Medicine University of Glasgow Glasgow UK; ^5^ Strathclyde Institute of Pharmacy and Biomedical Sciences University of Strathclyde Glasgow UK

**Keywords:** clinical medicine, diabetes, metabolic disease

## Abstract

**Introduction:**

The regulated delivery of the glucose transporter GLUT4 from intracellular stores to the plasma membrane underpins insulin‐stimulated glucose transport. Insulin‐stimulated glucose transport is impaired in skeletal muscle of patients with type‐2 diabetes, and this may arise because of impaired intracellular trafficking of GLUT4. However, molecular details of any such impairment have not been described. We hypothesized that GLUT4 and/or levels of proteins involved in intracellular GLUT4 trafficking may be impaired in skeletal muscle in type‐2 diabetes and tested this in obese individuals without and without type‐2 diabetes.

**Methods:**

We recruited 12 participants with type‐2 diabetes and 12 control participants. All were overweight or obese with BMI of 25–45 kg/m^2^. Insulin sensitivity was measured using an insulin suppression test (IST), and vastus lateralis biopsies were taken in the fasted state. Cell extracts were immunoblotted to quantify levels of a range of proteins known to be involved in intracellular GLUT4 trafficking.

**Results:**

Obese participants with type‐2 diabetes exhibited elevated fasting blood glucose and increased steady state glucose infusion rates in the IST compared with controls. Consistent with this, skeletal muscle from those with type‐2 diabetes expressed lower levels of GLUT4 (30%, *p* = .014). Levels of Syntaxin4, a key protein involved in GLUT4 vesicle fusion with the plasma membrane, were similar between groups. By contrast, we observed reductions in levels of Syntaxin16 (33.7%, *p* = 0.05), Sortilin (44%, *p* = .006) and Sorting Nexin‐1 (21.5%, *p* = .039) and −27 (60%, *p* = .001), key proteins involved in the intracellular sorting of GLUT4, in participants with type‐2 diabetes.

**Conclusions:**

We report significant reductions of proteins involved in the endosomal trafficking of GLUT4 in skeletal muscle in obese people with type 2 diabetes compared with age‐ and weight‐matched controls. These abnormalities of intracellular GLUT4 trafficking may contribute to reduced whole body insulin sensitivity.

## Graphical abstract

Impaired GLUT4 intracellular trafficking may underly defective muscle insulin‐stimulated glucose transport and hypothesised that levels of key proteins involved in intracellular GLUT4 trafficking are altered in type 2 diabetes. We observed that levels of both GLUT4 and proteins involved in GLUT4 endosomal trafficking are reduced in people with Type‐2 diabetes. Our data are consistent with aberrant GLUT4 intracellular sorting in people with type 2 diabetes and suggest that this may contribute to reduced whole body glucose disposal.

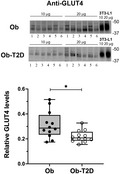

## 1 INTRODUCTION

Insulin resistance is an impaired cellular, tissue and whole‐body response to insulin and is one of the main underlying pathophysiological mechanisms leading to type‐2 diabetes (T2D). Insulin resistance occurs when increasing levels of insulin are required to exert a biological effect in the target tissues, and in particular adipose tissue and skeletal muscle. Insulin resistance can be present for many years before the development of T2D,^1^ hence, there is considerable interest in understanding the pathophysiological mechanisms that contribute to insulin resistance.

Insulin‐stimulated peripheral glucose dispersal is mediated primarily by skeletal muscle and the facilitative glucose transporter, GLUT4.^2^ Skeletal muscle is responsible for ~85% of whole‐body glucose disposal when quantified using the hyperinsulinaemic euglycaemic clamp.^3^ Individuals with T2D have, on average, a 50% reduction in whole‐body glucose disposal by this method.^4^ In the absence of insulin, GLUT4 is intracellularly sequestered in a population of vesicles, usually known as the GLUT4 storage compartment (GSC). In response to insulin a subset of these vesicles, referred to as insulin‐responsive vesicles (IRVs), redistribute to the cell surface where they dock and fuse, driving large increases in cell surface GLUT4 levels and increases in glucose transport into cells.^2^, ^5^, ^6^ The pathophysiological mechanism underlying insulin resistance in adipose tissue is reduced expression of GLUT4,^7^, ^8^ but the contributing mechanisms in skeletal muscle remain less clear.^9^, ^10^

There are conflicting reports regarding changes in GLUT4 levels in skeletal muscle associated with T2D.^10^ Dohm et al.^11^ suggested that GLUT4 levels were reduced in skeletal muscle of people with T2D. This has been supported by more recent studies, including a sensitive analysis of GLUT4 levels in different fibre types.^12^, ^13^ Lower skeletal muscle GLUT4 content also predicted impaired insulin sensitivity in chronic heart failure patients.^14^ However, other studies have suggested that GLUT4 levels are either unchanged^15^, ^16^, ^17^, ^18^ or only modestly reduced in T2D.^19^ Interestingly, Kahn et al.^20^ demonstrated a reduction in GLUT4 mRNA in streptozotocin induced mouse models of T2D, however, the observed insulin resistance preceded the reduction in GLUT4 mRNA suggesting that there are other contributing abnormalities.

Kelley et al.^21^ demonstrated that glucose transport and insulin stimulated GLUT4 translocation were attenuated in skeletal muscle of individuals with diabetes. There is evidence to suggest an impairment in GLUT4 trafficking in human skeletal muscle which may contribute to the observed insulin resistance in these tissues^10^: Insulin resistance was associated with aberrant intracellular GLUT4 sorting to a denser membrane compartment than is the case in control subjects; this abnormal subcellular distribution of GLUT4 was accompanied by reduced GLUT4 translocation following in vivo insulin stimulation.^18^ This led to the hypothesis that impaired insulin‐stimulated glucose transport arises from an impairment in GLUT4 trafficking into GSC which manifests as an accumulation of GLUT4 in a dense membrane compartment(s) from which insulin is unable to recruit GLUT4 to the cell surface.^18^ Neither the nature of this dense compartment nor the alterations in trafficking machinery that give rise to this altered trafficking are known. Furthermore, although reduced GLUT4 levels in skeletal muscle of obese patients has been reported,^22^ whether differences in skeletal muscle GLUT4 levels are present in obese patients with diabetes compared with obese non‐diabetic patients has not yet been addressed.

GLUT4 trafficking in response to insulin is a complex, tightly regulated process.^23^ In the absence of insulin, GLUT4 is sequestered within intracellular vesicles, collectively known as the GSC. A subset of these, IRVs, translocate to and fuse with the plasma membrane, delivering GLUT4 to the cell surface in response to insulin.^5^, ^6^, ^23^ Recent research has identified a range of proteins which regulate intracellular GLUT4 trafficking, including members of the SNARE family, components of the retromer complex and Sortilin—a member of the VPS10‐family of sorting receptors.^5^, ^24^, ^25^ Using cell lines in which expression or function of these molecules is impaired, clear evidence for a role of these endosomal trafficking proteins in the control of GLUT4 sorting to GSC/IRVs has been provided.^5^, ^26^, ^27^, ^28^, ^29^, ^30^ While useful for the identification of the mechanism of GLUT4 trafficking, whether these proteins are altered in T2D remains untested in human populations.

We hypothesized that GLUT4 and/or levels of proteins involved in sorting GLUT4 into the GSC may be impaired in skeletal muscle of patients with diabetes and have tested this in obese people with and without T2D characterized using an insulin suppression test (IST). Consistent with previous studies,^11^, ^12^, ^13^ we observed a modest decrease in GLUT4 levels in skeletal muscle in people with T2D compared with obese non‐diabetic controls. Accompanying this, we observed significant decreases in Syntaxin‐16 (Sx16) and sortilin levels: proteins known to mediate GLUT4 sorting into GSC,^5^ and selective decreases in members of the retromer family of endosomal sorting proteins.^31^ In contrast, levels of Syntaxin‐4 (Sx4) and VPS35 were unaffected. These data are consistent with the hypothesis that aberrant GLUT4 intracellular sorting is present in people with T2D and suggests that impaired delivery of GLUT4 into the GSC may contribute to reduced peripheral glucose disposal.

## 2 METHODS

### 2.1 Recruitment

All participants gave written informed consent. The study was approved by the West of Scotland Research Ethics Committee and performed in keeping with the Declaration of Helsinki at Glasgow Royal Infirmary, UK between March 2018 and February 2019. Participants were recruited by advertising in newspapers, and through NHS research databases including SHARE, Scottish Diabetes Research Network and Scottish Primary Care Research Network. Twelve participants with T2D (Ob‐T2D) and 12 control participants (Ob) matched for age and weight were recruited. All participants were white European males, had a BMI of 25–45 kg/m^2^, no personal history of cardiovascular disease and had normal liver, renal and thyroid function. In addition, those with T2D were managed by lifestyle or metformin only and had an HbA1c between 48 and 86 mmol/mol.

### 2.2 Assessment of insulin sensitivity

All participants attended the research facility for an initial screening visit involving written consent and baseline investigations (Table 1). Control participants underwent oral glucose tolerance tests to exclude underlying and undiagnosed impaired glucose tolerance or T2D. If all criteria were met, participants proceeded to an insulin suppression test (IST) with collection of muscle biopsies.

**TABLE 1:** Participant characteristics

	Ob Participants without T2D^a^ (*n* = 12)	Ob Participants with T2D (*n* = 12)
Age (years)	50.8 ± 3.8	57.3 ± 1.5
BMI (kg/m^2^)	30.0 ± 0.8	33.8 ± 1.7
Systolic BP (mmHg)	126.8 ± 4.5	148.7 ± 4.0
Diastolic BP (mmHg)	81.5 ± 2.9	89.2 ± 1.8
Fasting glucose (mmol/l)	5.0 ± 0.1	9.3 ± 0.9*
HbA1c (mmol/mol)	n/a	60.7 ± 3.4
Cholesterol (mmol/l)	5.0 ± 0.3	3.8 ± 0.22

*Note*: Baseline characteristics of participants are presented as mean ± SD. HbA1C was not measured in those without T2D. *Indicates a significant difference compared with participants without T2D, *p* < .0001.

^a^ BY OGTT.

Measurements of insulin resistance in clinical research have been used for many years to define the degree of insulin resistance or insulin sensitivity. This has often been in the form of the hyper‐insulinemic euglycemic clamp, however, the insulin suppression test (IST) has been shown to be an effective, well tolerated and highly comparable alternative.^32^ The IST involves continuous infusions of glucose and insulin followed by measurements of steady state plasma insulin and steady state plasma glucose (SSPI and SSPG respectively). This test was used to quantify insulin‐sensitivity in our cohort.

**FIGURE 1:**
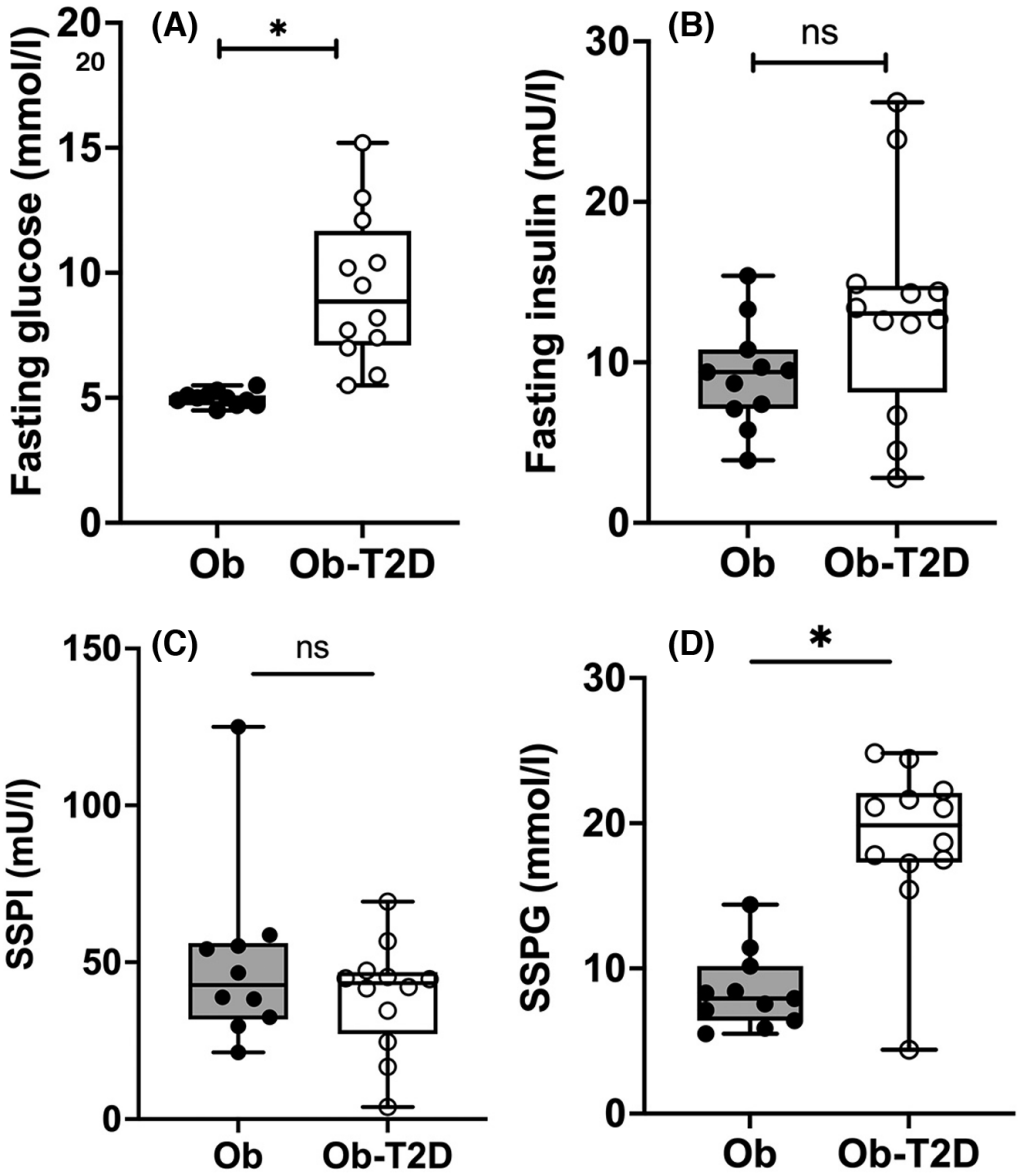
IST insulin sensitivity. Plasma glucose and plasma insulin were obtained in the fasted state prior to commencing the IST and compared between obese participants with and without T2D. Fasting results are shown in panels A and B. Plasma insulin and glucose samples were obtained every 10 min from 150 to 180 min of the IST and the mean calculated to determine steady state levels (SSPG and SSPI) which are shown in C and D respectively. Individual values are presented along with the mean and standard deviation (S.D). Panels A and B represent 12 people with T2D (Ob‐T2D) and 12 people without T2D (Ob); Panels C and D are from 11 participants without T2D as one patient withdrew after muscle biopsy but before the IST (see Methods.) Statistical analysis was performed using a two‐tailed unpaired *t*‐test, with significant differences indicated by *p < .0001.

Insulin suppression tests were performed with continuous infusions of glucose, insulin and octreotide with infusion rates calculated according to body surface.^32^ After muscle biopsies were obtained, infusion of 20% (w/v) dextrose, insulin and octreotide were commenced at 267 mg/m^2^/min, 32 mU/m^2^/min and 0.27 μg/m^2^/min respectively. Blood sampling was performed every 30 min for 150 min to measure point of care glucose to ensure the glucose remained within safe limits. From 150 until 180 min, plasma insulin and plasma glucose samples were obtained every 10 min. The four values obtained from 150–180 min represented the steady state plasma insulin and steady state plasma glucose (SSPI and SSPG respectively).^32^ Once final blood had been obtained at 180 minutes, infusions were discontinued, and participants provided with a carbohydrate‐based meal and point of care glucose checked 30 minutes later to ensure stability prior to discharge. One participant in the control group developed a hematoma after the muscle biopsy and was withdrawn prior to completing the IST, but their data were included in all figures except Figure 1C,D (SSPI and SSPG datasets).

### 2.3 Muscle biopsy

Muscle biopsies were performed at baseline in the fasted state. The site was identified by measuring 20 cm above the patella and projecting a line laterally on the lateral aspect of the vastus lateralis muscle. Under sterile conditions, the area was cleaned, and the skin infiltrated with 5 ml of 1% (w/v) lidocaine. Once the anaesthetic effect had occurred, a small incision around 0.5 cm was made at the biopsy site using a scalpel. Five samples were then taken each weighing approximately 50 mg using a Bard automatic biopsy needle. These were snap frozen in liquid nitrogen before being stored at −80°C.

### 2.4 Generation of skeletal muscle lysates

Skeletal muscle samples were thawed on ice and homogenized through a 19 G needle in 200 μl of ice‐cold lysis buffer (25 mM Tris–HCl (pH 7.4), 50mMNaF, 100 mM NaCl, 1 mM sodium vanadate, 5 mM EGTA, 1 mM EDTA, 1% (v/v) Triton X‐100, 10 mM sodium pyrophosphate, 0.27 M sucrose, Complete™ Protease inhibitor cocktail tablets (1tablet/10 ml), 0.1% (v/v) 2‐mercaptoethanol). Samples were subsequently centrifuged at 13,000 *g* for 10 min and the supernatant combined with Protein G‐Sepharose for 1 h with rotation at 4°C to remove any IgG from blood present in the samples. After 1 h, samples were centrifuged at 16,000*g* for 30 seconds, the supernatant removed and stored at −80°C.

### 2.5 3 T3‐L1 cell culture and lysate preparation

3 T3‐L1 adipocytes, purchased from the American Tissue Culture Collection (ATCC Cat# CCL‐92.1, RRID:CVCL_0123) were grown, maintained and differentiated exactly as outlined previously.^24^ A single batch of cells were used at 10‐day post‐differentiation to generate a lysate, as described.^33^ Enough of this lysate was prepared so as be included on all gels analysed and thus act as a quantitative comparison for all experiments.

### 2.6 Immunoblotting

Samples were thawed and combined with Laemmli sample buffer and heated to 65°C for 10 min prior to SDS‐PAGE and immunoblotting. Blots were visualized using LICOR infra‐red fluorescence detection and quantified using company proprietary software. The antibodies used in this study were anti‐GLUT4 (Thermo Fisher Scientific Cat# PA1‐1065, RRID:AB_2191454), anti‐Sx16 (Synaptic Systems Cat# 110162, RRID:AB_887799), anti‐Sx4 (Synaptic Systems Cat# 110042, RRID:AB_887853), anti‐SNX1 (Proteintech Cat# 10304‐1‐AP, RRID:AB_2192217; the band at 70 kDa present in both 3 T3‐L1 adipocytes and skeletal muscle was quantified), anti‐SNX27 (Proteintech Cat# 16329‐1‐AP, RRID:AB_10888628), anti‐VPS35 (Proteintech Cat# 10236‐1‐AP, RRID:AB_2215216) and anti‐MAPK (Santa Cruz Biotechnology Cat# sc‐514,302, RRID:AB_2571739).

### 2.7 Inter‐gel comparisons and statistical analysis

Muscle samples were assessed by immunoblotting and compared with equivalent protein levels of 3 T3‐L1 adipocytes. A single preparation of 3 T3‐L1 adipocyte lysate was used on all immunoblots for every antibody, allowing comparisons to be made between different immunoblots of muscle lysates, and between control and T2D groups. Ten and 20 μg of muscle biopsy and 3 T3‐L1 adipocyte lysate was loaded on each gel to confirm linearity of detection. All biopsy samples were immunoblotted three times using each antibody, with results normalized to levels of the corresponding protein in 3 T3‐L1 adipocytes to allow statistical comparisons between groups and comparison of signals across multiple gels. Given that we have no information on how metabolic enzymes (such as GAPDH) or scaffold proteins (such as actin) may differ between samples, we decided that comparison with a constant external standard (3 T3‐L1 lysate) was less likely to be the subject of a systematic error and would facilitate inter‐gel and inter‐group comparisons. Triplicate replicates of all samples were performed to mitigate against intra‐gel transfer artefacts.

Statistical testing was performed with GraphPad Prism 7. In all figures, each data point represents a single participant. For all immunoblot figures, each data point is the mean of three technical replicates of each sample. Box and whisker plots of the immunoblot data are presented. Comparison between control and T2D samples was by two sample *t*‐test. *p* values are provided in each figure legend. Each group consisted of *n* = 12 participants.

## 3 RESULTS

### 3.1 Participant demographics

Twelve obese participants with T2D (Ob‐T2D) and 12 obese (Ob) controls completed the study. The demographics of the participants are detailed in Table 1. Of the 12 Ob‐T2D participants, 10 were prescribed metformin and the remaining two participants were managed by lifestyle factors only. Seven Ob‐T2D participants had a known diagnosis of hypertension and were prescribed anti‐hypertensives including ACE inhibitors such as ramipril, calcium channel blockers such as amlodipine, and one participant was prescribed the thiazide diuretic, bendroflumethiazide. Ten out of 12 Ob‐T2D were prescribed statin therapy compared with one in the Ob group and had lower serum cholesterol levels than those without T2D (3.8 ± 0.22 vs. 5.0 ± 0.3 mmoL/L). Medications in participants without T2D included proton pump inhibitors including lansoprazole, anti‐depressants such as sertraline and antihistamines including cetirizine. All participants had normal renal function, liver function and thyroid function.

### 3.2 Assessment of insulin sensitivity

Fasting glucose was significantly higher in the Ob‐T2D participants than in the Ob (non‐diabetic) group (9.3 ± 0.9 vs. 5.0 ± 0.1 mmoL/L, *p* < .0001; Figure 1A). Ob‐T2D participants had numerically but not statistically higher fasting insulin concentrations compared with controls (13.2 ± 4.0 vs. 9.2 ± 1.9 mU/L, *p* = .09; Figure 1B). SSPI results were consistent between groups as expected given infusion of insulin according to the body surface area (48.8 ± 14.8 vs. 38.0 ± 10.1 mU/L, *p* = .3; Figure 1C) and suppression of endogenous production with values are around five‐fold higher than baseline fasting plasma insulin. As expected, SSPG results were significantly higher in Ob‐T2D than in Ob non‐diabetic participants (18.9 ± 1.7 vs. 8.5 ± 1.6 mmoL/L, *p* < .0001; Figure 1D). Assuming similar insulin clearance, the difference in SSPG between groups demonstrates that Ob‐T2D participants have a reduction in peripheral glucose uptake, indicating greater insulin resistance than the control subjects despite comparable SSPI levels, consistent with the pathophysiology of T2D.

### 3.3 GLUT4 levels are reduced in obese patients with T2D

**FIGURE 2:**
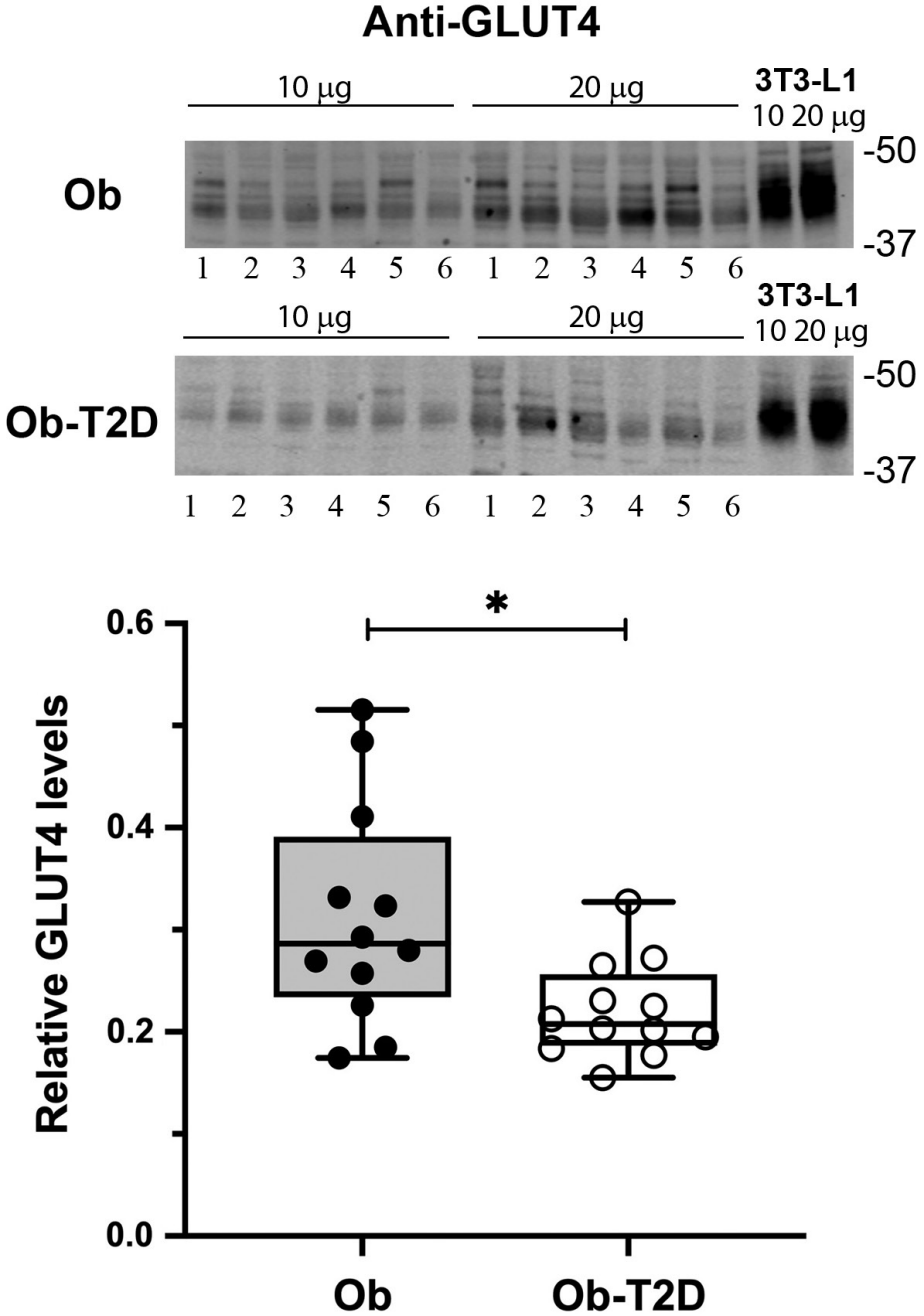
GLUT4 levels in skeletal muscle from people with and without T2D. Vastus lateralis skeletal muscle biopsy samples were obtained from study participants in the fasted state. Samples were subjected to immunoblotting. (A) shows representative immunoblots from six participants without T2D (Ob) and six with T2D (Ob‐T2D) (labelled 1–6) for GLUT4, respectively, loaded as two separate amounts, 10 and 20 μg, along with similar amounts of 3 T3‐L1 adipocyte lysate for comparison. (B) is a box and whisker plot comparing the ratio of skeletal muscle GLUT4 to 3 T3‐L1 GLUT4 levels from all subjects with (Ob‐T2D) and without (Ob) T2D (*n* = 12) repeated three times on separate gels. Each point is the mean of triplicate determinations and thus represents an individual subject. Statistical analysis was performed using an unpaired *t*‐test. *p = .014

Skeletal muscle samples were first immunoblotted for GLUT4 (Figure 2). GLUT4 levels were significantly reduced by 30% in the Ob‐T2D group compared with Ob participants (*p* = .014); this observation is consistent with previous studies^11^, ^12^, ^13^ which also found similar reductions in GLUT4 in skeletal muscle from people with T2D.

### 3.4 Syntaxin 16 but not Syntaxin 4 levels are reduced in patients with T2D

**FIGURE 3:**
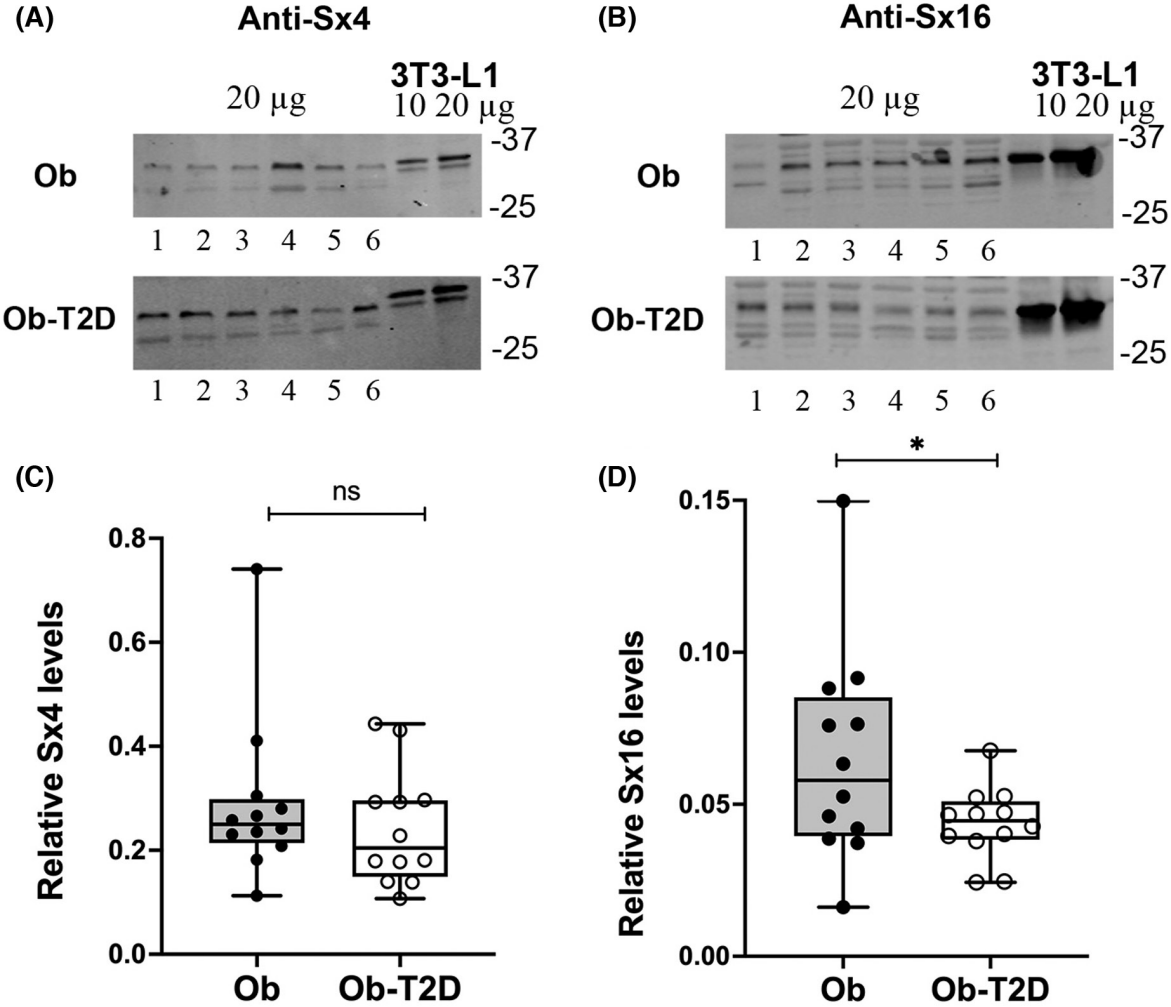
Syntaxin levels in skeletal muscle from people with and without T2D. Skeletal muscle samples (20 μg) from 6 participants (numbered 1–6) in each group were immunoblotted for Sx4 (panel A) and Sx16 (panel B) as indicated. (C and D) are box and whisker plots of the ratio of skeletal muscle Sx4 and Sx16, respectively, to 3 T3‐L1 levels in 12 people with and 12 people without T2D. Each point is the mean of triplicate determinations and thus represents an individual subject. Statistical analysis was performed using an unpaired *t*‐test (*p = .05; n.s., not significantly different, *p* = .37).

We chose to examine levels of Sx4 and Sx16 which are involved in fusion of GLUT4 storage vesicles with the cell surface and recycling GLUT4 away from the cell surface and back into storage vesicles, respectively (see^5^, ^6^ for reviews). The results are shown in Figure 3. There were no statistically significant differences in levels of Sx4 between those with and without T2D (*p* = .37). However, levels of Sx16 were reduced by an average of 34% in Ob‐T2D participants compared with Ob non‐diabetic participants (*p* = .05). The knockdown of Sx16 in 3 T3‐L1 adipocytes has been shown to result in both reduced total levels of GLUT4 and impaired sorting of GLUT4 from the endosomal system into GSC/IRV.^26^, ^34^ Our data are therefore the first to suggest that a similar defect may be present in T2D. We therefore focussed on other proteins that are known to affect endosomal GLUT4 trafficking.

### 3.5 Endosomal sorting protein levels in patients with T2DM

**FIGURE 4:**
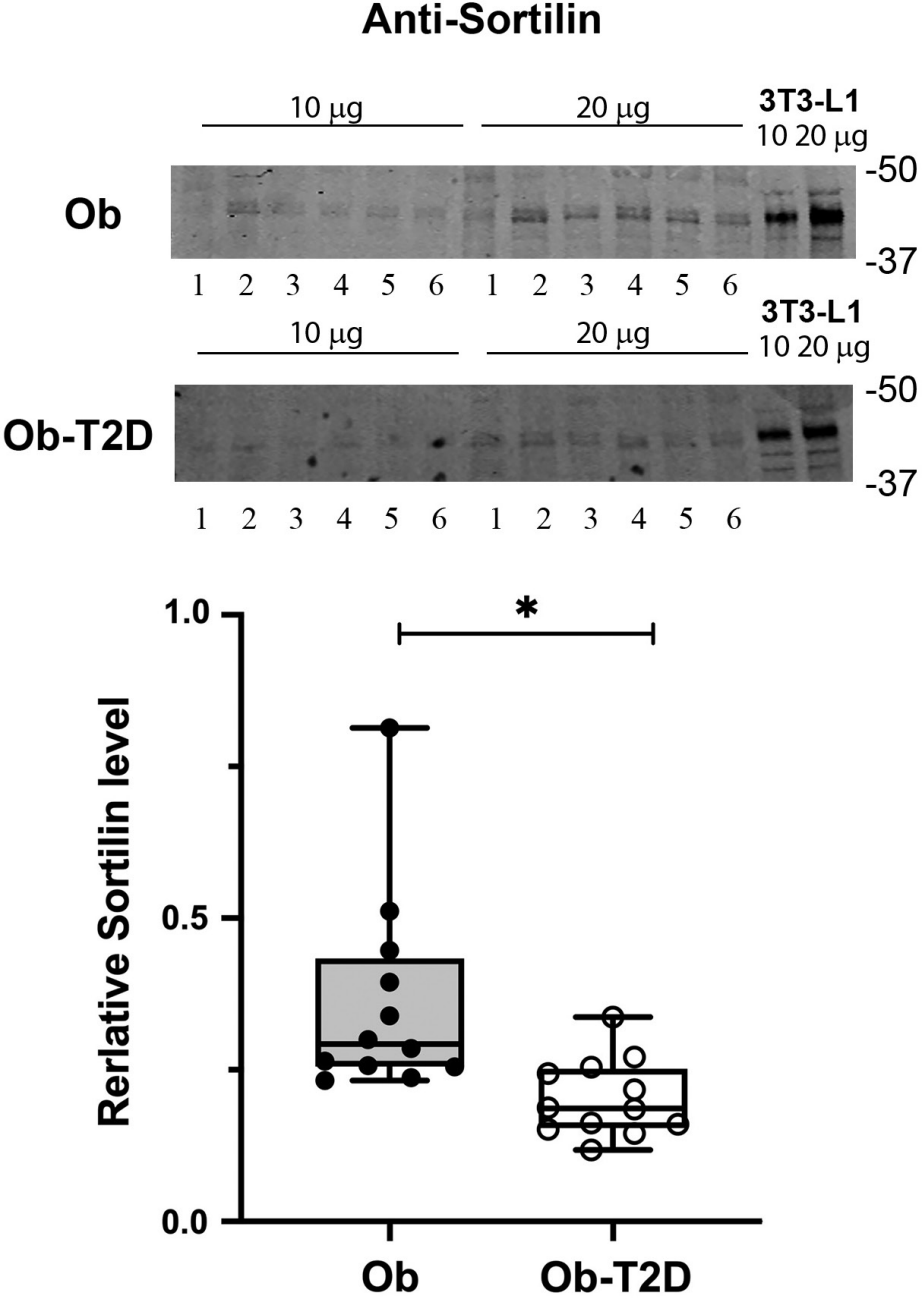
Levels of Sortilin in skeletal muscle from people with and without T2D. Skeletal muscle samples (20 μg) from 6 participants (numbered 1–6) in each group were probed for Sortilin as described in Figure 2. A typical immunoblot is shown in A and quantification of the entire dataset is presented in panel B as a box and whisker plot. Each point is the mean of triplicate determinations from each subject. Statistical analysis was performed using an unpaired *t*‐test, significant differences are shown by *p = .006).

Sortilin plays a role in sorting GLUT4 from the endosomal system into GSC/IRV in adipocytes,^27^, ^35^ and thus, we examined sortilin in our samples (Figure 4). Levels of sortilin are reduced on average by 44% in the skeletal muscle of Ob‐T2D participants (*p* = .006; Figure 4). This is a potentially significant finding as sortilin is thought to be required for formation of IRV, at least in murine 3 T3‐L1 adipocytes.

**FIGURE 5:**
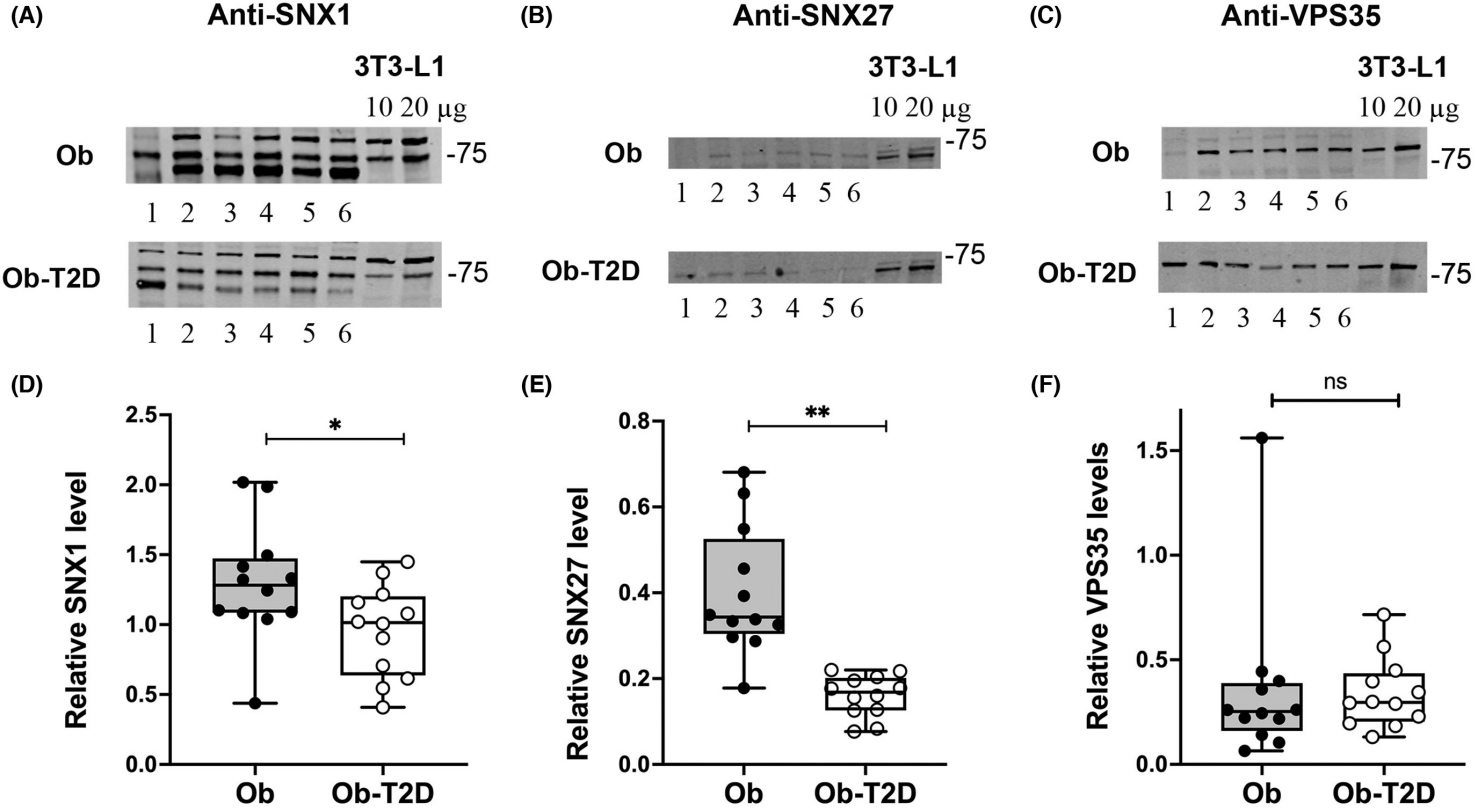
Sorting nexins in skeletal muscle from people with and without T2D. Skeletal muscle samples (20 μg) from 6 participants (numbered 1–6) in each group were probed using anti‐SNX1 (panels A), anti‐SNX27 (panels B) or anti‐Vps35 antibodies (panel C). Typical blots (A–C) are shown, together with box and whisker plots of the entire data set (panels D–F). Each point is the mean of triplicate determinations from each study subject. Statistical significance between the groups is indicated by *p = .039, **p = .0001; n.s., not significantly different.

The retromer complex plays a key role in the recycling of proteins between endosomes and the trans Golgi network.^31^ A role for retromer in controlling GLUT4 trafficking between endosomal and lysosomal compartments has been suggested by studies using cultured cells.^29^, ^30^, ^36^ Here, we quantified the levels of sorting nexins 1 and 27 (SNX1 and SNX27) and vacuolar protein‐sorting 35 (VPS35; Figure 5). Both SNX1 and SNX27 were significantly reduced in the skeletal muscle of Ob‐T2D subjects compared with those without T2D (by 22%, *p* = .039 and 60%, *p* = .0001, respectively). By contrast, VPS35 levels were not significantly altered.

## 4 DISCUSSION

It is recognized that the pathogenesis of insulin resistance in skeletal muscle is due to impaired GLUT4 trafficking but the exact cause for this remains unclear.^9^, ^23^, ^37^, ^38^, ^39^, ^40^, ^41^ Here, we sought to compare skeletal muscle of obese people with T2D and obese non‐diabetic controls to define whether changes in GLUT4 levels and/or alterations in key trafficking components were associated with insulin resistance in the obese T2D population. As expected, Ob‐T2D participants were more insulin resistant than those without T2D as demonstrated by higher SSPG despite comparable SSPI levels by IST, demonstrating a reduction in whole body glucose consumption (Figure 1).

We observed significant reduction in levels of GLUT4 in Ob‐T2D participants compared with Ob controls without T2D (Figure 2). While there is evidence in human skeletal muscle studies that GLUT4 expression is unaffected T2D,^9^, ^15^, ^16^, ^18^, ^21^ others have reported reductions in skeletal muscle GLUT4 levels similar to those reported here.^11^, ^12^, ^13^ The area is well discussed in a recent review.^10^ Furthermore, reduction in skeletal muscle GLUT4 levels in obesity have also been reported.^22^ Limitations to studies in this area, including the present one, are the small numbers of participants that can readily be studied using invasive techniques and heterogeneity between individuals. Kahn et al.^16^ studied 30 nonobese participants with insulin dependent diabetes who were insulin resistant with a mean age of 34 years; Garvey et al.^18^ studied lean and obese controls, as well as obese people with impaired glucose tolerance and T2D; and finally, Pedersen et al studied 17 people with T2D, some of whom were newly diagnosed and treatment naive, compared with lean and obese controls.^15^ This variation in age, treatment and duration of T2D makes direct comparisons difficult, especially when T2D is already associated with significant heterogeneity between individuals.^42^ Indeed, Pedersen et al.^40^ comment on the variability observed in expression of GLUT4 between groups which is likely to be an important factor. Although every effort was made to match our study participants, BMI was numerically higher in those with T2D and they were not completely matched for other factors that may have influenced insulin sensitivity, including duration of T2D, blood pressure and statin use/cholesterol levels (Table 1). Nevertheless, we have attempted to compare participants with similar levels of obesity in the presence or absence of diabetes to identify any specific changes associated with T2D. Consistent with published work,^11^, ^12^, ^13^ we observed a 30% reduction in skeletal muscle GLUT4 levels in patients with T2D (Figure 2).

In addition to glucose transporters, various other key proteins that are involved in glucose transport were analysed in these skeletal muscle samples. Firstly, levels of the t‐SNARE Sx4 thought to be a major participant in the fusion of GLUT4‐containing vesicles^5^, ^6^, ^23^ with the cell surface were found to be unchanged between those with and without T2D (Figure 3). By contrast, the intracellular SNARE Sx16 was significantly reduced in T2D (Figure 3). Sx16 is a t‐SNARE involved in the sorting of GLUT4 from recycling endosomes into the GSC.^26^, ^34^ Previous studies have shown that either knockdown or inhibition of Sx16 in cultured adipocytes impairs insulin‐stimulated glucose transport and GLUT4 translocation as well as causing a 30% reduction in total GLUT4 levels.^26^ This reduction in GLUT4 is thought to reflect aberrant trafficking of the transporter into degradative pathways rather than into GSC/IRV.^24^ The data observed here in T2D are strikingly similar, prompting us to consider whether other proteins known to regulate GLUT4 endosomal trafficking were similarly affected.

Sorting of GLUT4 from the recycling endosomal system into the GSC involves a complex suite of proteins acting at different stages of the GLUT4 trafficking itinerary; while may such proteins have been identified, a unifying model remains elusive and no study has systematically examined whether changes in the levels of these proteins accompany diabetes.^5^, ^6^, ^27^ Sortilin is expressed in both adipocytes and skeletal muscle and is essential for the formation of the GSC; depletion of sortilin blocks delivery of GLUT4 into GSC and increases its degradation.^29^, ^35^ Sortilin mRNA and protein expression are reduced in adipose tissue and skeletal muscle of mouse models of both obesity and diabetes^43^ and sortilin levels are regulated by glucose depravation in rodent skeletal muscle,^44^ but to the best of our knowledge, there are no studies of human muscle in which sortilin levels have been quantified. We observed a 44% reduction in sortilin levels in Ob‐T2D participants compared with controls (Figure 4). By analogy with studies in cultured cells,^35^, ^45^ this reduction could attenuate the storage of GLUT4 into the GSC and thus redirect GLUT4 into insulin insensitive cell compartments, or degradative pathways resulting reduction in GLUT4 (Figure 2) and impaired insulin sensitivity (Figure 1).

The role of retromer complexes in sorting and trafficking transmembrane proteins within endosomes is well established.^46^ The retromer complex consists of VPS35 (in combination with VPS26 and VPS29) and sorting nexins, for example SNX1 and SNX27. Knockdown of SNX 27 or VPS35 in human or rodent adipocytes decreased the stability of sortilin and GLUT4, impaired GLUT4 sorting and decreased insulin‐stimulated glucose transport.^29^, ^30^, ^36^ Others have suggested that an interaction between Vps35 and sortilin is regulated by insulin^47^ and thus we hypothesized that retromer levels may be altered in T2D. We observed a significant reduction in SNX1 and SNX27 in skeletal muscle of people with T2D, compared with controls, but unchanged protein expression of VPS35. In cultured adipocytes, both SNX1 and SNX27 have been shown to be involved in GLUT4 trafficking by regulating recycling of proteins via endosomes and demonstrate a degree of insulin stimulated translocation.^29^, ^48^ This reduction in sorting nexins in skeletal muscle of participants with T2D is a potentially exciting development and may point towards their role in not only GLUT4 trafficking but the pathogenesis of insulin resistance and T2D. It is interesting that the reductions are selective—VPS35 levels are unchanged, hinting at differential roles of SNXs in GLUT4 sorting. Future studies will be needed to address this point. Further in relation to this point, we observed no changes in levels of either Akt or ERK1/2, two key signalling kinases, between the groups (not shown).

Some limitations of this study, including incomplete matching for degree of obesity and concomitant treatment, should be acknowledged. We were unable to examine IRAP levels in these samples as a result of a cross‐reacting protein of similar molecular weight which prevented analysis. Similarly further studies were limited by a lack of availability of suitable reagents, for example to USP25,^5^, ^27^, ^36^ and limited amounts of biological samples. Future studies of other proteins, such as components of the Golgi by‐pass route controlled by CHC22^5^, ^49^ will be important and worthwhile goals. We note that ours is a relatively small sample size but feel that our comparison of obese patients with or without diabetes makes a useful contribution to the field. It will be of interest in the future to ascertain whether these observed effects are manifest at the level of transcriptional or post‐translational regulation.

In conclusion, this study has demonstrated significant changes in proteins involved in the endosomal trafficking of GLUT4 in skeletal muscle in Ob‐T2D participants compared to participants without T2D. These include Sx16, sortilin and sorting nexins—SXN1 and SNX27. With the caveat of incomplete matching, these findings suggest that multiple abnormalities can be detected at various stages of intracellular GLUT4 trafficking in T2D. These may re‐direct GLUT4 into dense membrane compartments or degradative pathways and therefore result in the reductions observed in levels of GLUT4 in skeletal muscle.

## AUTHOR CONTRIBUTIONS

**Rachel Livingstone:** Data curation (equal); methodology (equal); validation (equal); visualization (equal); writing – original draft (equal). **Nia J. Bryant:** Conceptualization (equal); writing – original draft (equal); writing – review and editing (equal). **James G. Boyle:** Funding acquisition (equal); project administration (equal); supervision (equal); writing – review and editing (equal). **John R. Petrie:** Funding acquisition (equal); investigation (equal); project administration (equal); writing – original draft (equal); writing – review and editing (equal). **Gwyn W. Gould:** Conceptualization (equal); data curation (equal); formal analysis (equal); funding acquisition (equal); investigation (equal); project administration (equal); supervision (lead); validation (equal); writing – original draft (equal); writing – review and editing (equal).

## FUNDING INFORMATION

This work was supported by an award from Novo Nordisk UK Research Foundation (to GWG, JRP and JGB), by a Chief Scientist Office NRS Career Research Fellowship (to JGB) and by NHS Greater Glasgow and Clyde Endowment Fund (to JGB and RL). The funders played no role in any aspect of the work.

## CONFLICT OF INTEREST

The authors declare they have no competing interests.

## Data Availability

The datasets generated during and/or analysed during the current study are available from the corresponding author on reasonable request.
